# Paraneoplastic pyoderma gangrenosum associated with adenocarcinoma of the rectosigmoid junction: a case report

**DOI:** 10.1186/s13256-019-2290-6

**Published:** 2019-12-07

**Authors:** Fousséni Alassani, Panawe Kassang, Efoe-Ga Amouzou, Boyodi Tchangai, Kossi Abossisso Sakiye, Tchin Darré, Bayaki Saka, Komla Attipou

**Affiliations:** 1 Department of Visceral Surgery, University Teaching Hospital of Lomé, University of Lomé, BP 1515 Lomé, Togo; 2 Department of Dermatology, University Teaching Hospital of Lomé, Lomé, Togo; 3 Department of General Surgery, University Teaching Hospital of Kara, Kara, Togo; 4 Department of Surgical Emergency, University Teaching Hospital of Lomé, Lomé, Togo; 5 Department of Pathology, University Teaching Hospital of Lomé, Lomé, Togo

**Keywords:** Paraneoplastic pyoderma gangrenosum, Adenocarcinoma, Rectosigmoid, Colonoscopy

## Abstract

**Introduction:**

Pyoderma gangrenosum is a rare, idiopathic, inflammatory, neutrophilic dermatitis characterized by sterile skin ulceration. It can be associated with an underlying pathology, especially inflammatory bowel disease and hematological malignancies. Its association with a malignant pathology in the context of a paraneoplastic syndrome is more commonly described in hematological malignancies, with solid tumors being rare.

**Case report:**

We report a case of a 39-year-old West African man with pyoderma gangrenosum that developed 6 months before the clinical expression of rectosigmoid junction cancer. The removal of the cancer resulted in the patient’s recovery.

**Conclusion:**

Recurrent pyoderma gangrenosum lesions may be the expression of colonic adenocarcinoma in paraneoplastic syndrome and require colonoscopy, especially in at-risk patients.

## Introduction

Pyoderma gangrenosum (PG) is a rare, idiopathic, inflammatory, neutrophilic dermatosis that is generally characterized by recurrent sterile skin ulceration, with rare extracutaneous involvement [1–3]. Because of its clinical polymorphism related to its different clinical forms, its similarity to other infectious or vascular skin disorders, and its nonspecific histological nature, PG is often a diagnosis of elimination [4]. It can be associated with underlying systemic diseases, the most common of which are inflammatory bowel disease, rheumatic diseases, and malignant blood diseases [5]. Research on the pathology associated with PG is therefore part of the management strategy, especially in the case of cancers for which paraneoplastic forms have been described [6]. Thus, since the first description of paraneoplastic PG in myeloproliferative cancers, few cases of paraneoplastic PG associated with a solid tumor have been described [5]. We report a case of PG associated with adenocarcinoma of the rectosigmoid junction.

This case is peculiar because of the occurrence of PG lesions 6 months before the onset of rectosigmoid junction cancer symptoms and their disappearance after tumor removal. This shows that PG may be the first manifestation of colorectal adenocarcinoma in paraneoplastic syndrome.

## Case presentation

A 39-year-old West African man who was a teacher was referred for weight loss and recent abdominal pain. He presented the following symptoms: generalized episodic abdominal pain of progressive onset that was relieved by diarrheal stools occasionally associated with rectal bleeding over the past 2 months. The patient had recurrent skin lesions of different ages on the lower limbs that had started to appear 6 months before the onset of abdominal pain. These lesions began with an inflammatory nodule that resolved within a few days with a pustule that ruptured quickly and spontaneously gave way to an ulceration whose diameter quickly reached several centimeters. The healing was spontaneous and left hypertrophic stigmata.

**Fig. 1:**
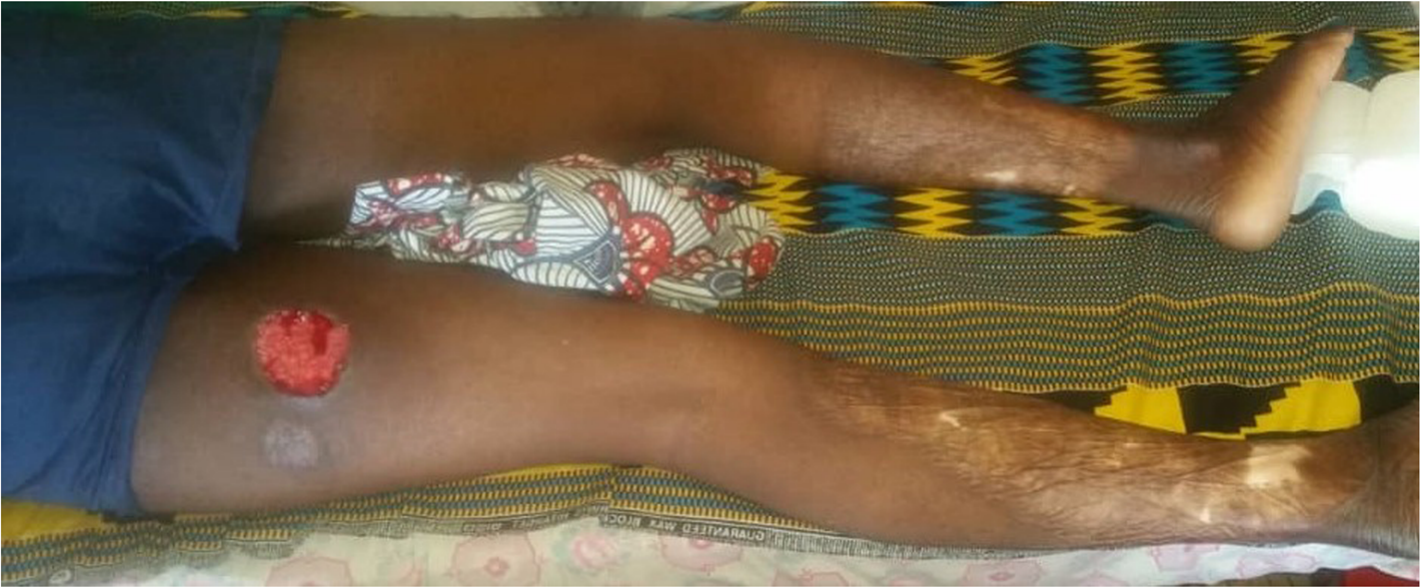
Scarring lesion and progressive ulceration

The patient was not a drinker or smoker and was of middle socioeconomic status. He had no personal or family medical history, especially cancer or blood or inflammatory disease. Upon admission, he had a body temperature of 37.4 °C, a pulse rate of 81 beats per minute, and a blood pressure of 110/85 mmHg. His physical examination revealed a moderate general condition, moderately colored conjunctiva, pain in the left iliac fossa, and hypogastrium without a palpated mass. The result of his rectal examination was normal. His dermatological examination demostrated objectified large, circumferential scarring lesions on both lower limbs and progressive ulceration on the posterior side of the left thigh (Fig. 1). His Glasgow Coma Scale score was 15; he had no sensory or motor deficit, and the result of examination of his cranial nerves was normal. Biopsy of the progressive lesion showed a suppurative and inflammatory central zone with ulceration and neutrophil infiltration. The results of cytobacteriological (aerobic and anaerobic culture) and fungal studies were negative.

**Fig. 2:**
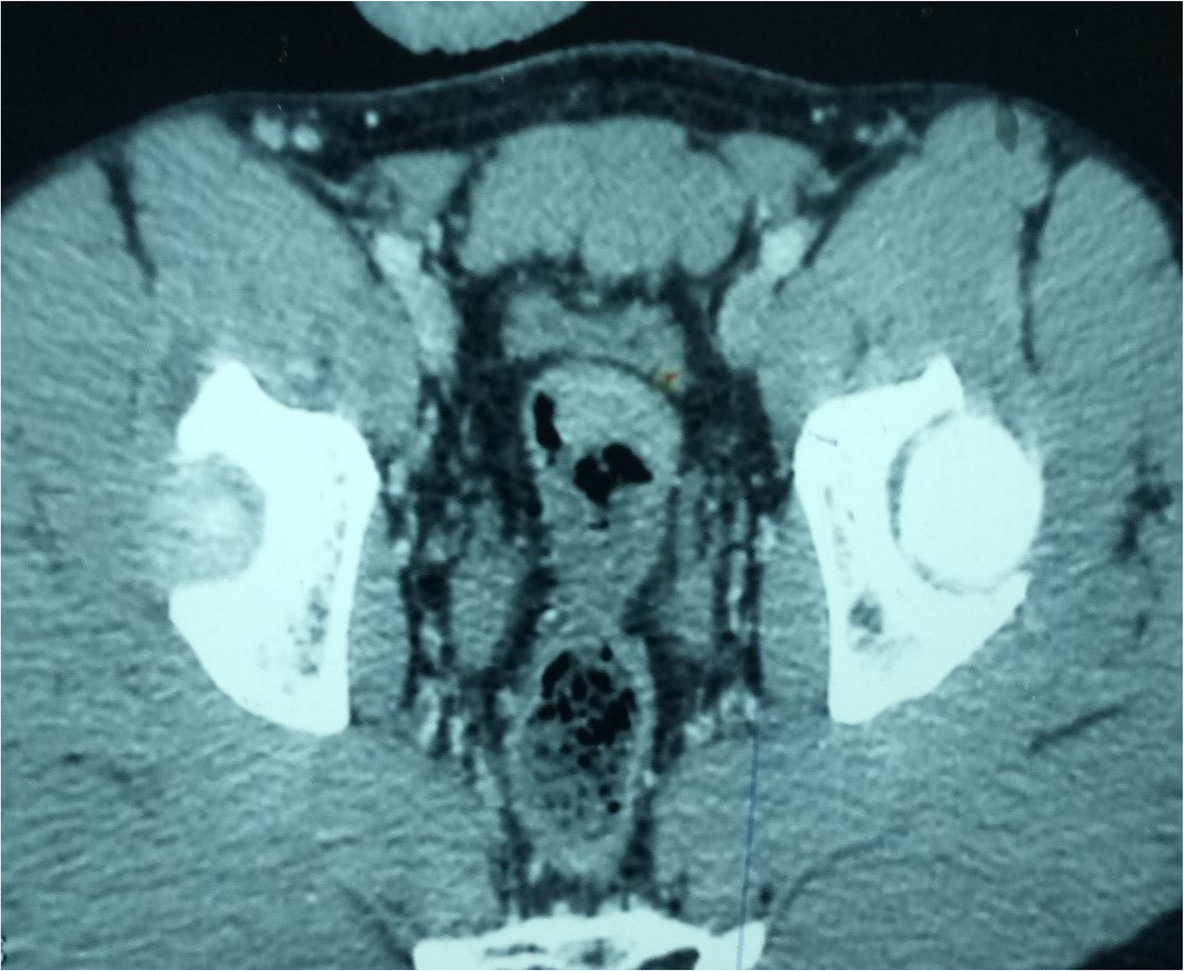
Computed tomographic scan showing the tumor

**Fig. 3:**
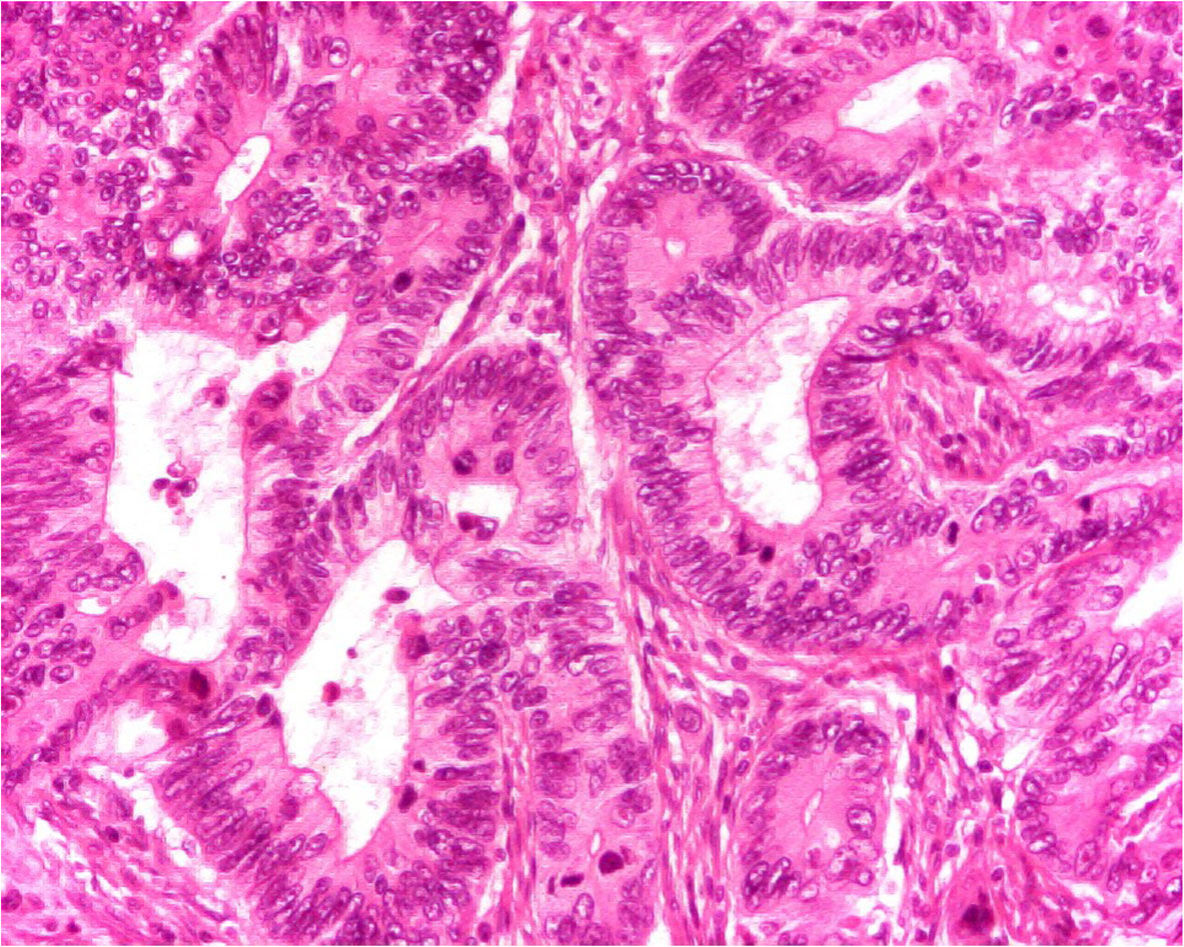
Histological image of the tumor

We concluded that these skin lesions were PG. Colonoscopy for the exploration of abdominal symptomatology revealed a necrotic and hemorrhagic budding lesion 20 cm from the anal margin, confirmed by contrast enema, which showed a narrowing of the lumen of the rectosigmoid junction. Biopsies taken during colonoscopy revealed an adenocarcinoma. An abdominopelvic computed tomographic scan revealed thickening of the rectosigmoid without associated metastases (Fig. 2). The results of preoperative checkup (rhesus grouping; complete blood count; prothrombin time; activated cephalin time; tests for uremia, creatinemia, and glycemia; chest x-ray; and electrocardiogram) and liver checkup (serum glutamic-oxaloacetic transaminase (SGOT), serum glutamate-pyruvate transaminase (SGPT), alkaline phosphatase, gamma glutamyltransferase (GGT), total and direct bilirubin) were normal. A median subumbilical laparotomy confirmed a stenosing tumor of the rectosigmoid junction. A rectosigmoid carcinologic excision was performed with immediate colorectal anastomosis. Regular bandages were placed on the PG lesions. Postoperative treatment was initiated with ciprofloxacin 500 mg and metronidazole 500 mg twice daily for 10 days and tramadol 50 mg three times daily for 5 days. The anatomopathological examination of the surgical specimen (using hematoxylin and eosin-stained sections) confirmed the diagnosis of adenocarcinoma of the rectosigmoid junction (Fig. 3). The tumor was classified as pT2N0M0 with negative excision margins. There were no postoperative complications, and the patient was discharged on postoperative day 7. No adjuvant treatment was performed. Thirteen months after the operation, all the skin lesions had healed without reemergence of new lesions, and the patient did not have metastases or tumor recurrence.

## Discussion

Our patient presented with the association of PG and adenocarcinoma of the rectosigmoid junction, which is rarely described. However, this case report is distinguished by the appearance of PG lesions before the clinical expression of cancer and their disappearance after tumor excision.

PG is a dermatosis that can occur in isolation. However, in more than 75% of cases, it is associated with an underlying pathology, including inflammatory bowel disease; endocrine, hematological, and rheumatological disorders; and nonhematological neoplasia [4]. Few cases of PG related to colorectal cancer have been described. Those that have been reported have been either a single skin lesion located on the trunk or the upper limbs for some [7–9] or ulcerative colitis that was associated with rectal cancer, which made a direct causal relationship between rectal cancer and PG questionable [10]. Cailhol *et al.* [11] described a cutaneous (lower limb location) and extracutaneous (lung location) PG with secondary appearance during neoplastic progression after the first line of chemotherapy. Our patient’s case is distinguished from previous cases by its premature appearance before the diagnosis of adenocarcinoma without any other associated disease, the multiple lesions of PG, and their location in the lower limbs. The appearance of the first PG lesions 6 months before the cancer diagnosis, their recovery without reappearance of new lesions 4 months after resection of the tumor, and the absence of any other underlying condition made us consider its paraneoplastic character. Our patient’s case is similar to the one described by You *et al.* [5], which makes our patient’s case, to our knowledge, the second observation of paraneoplastic PG on colorectal adenocarcinoma appearing before any clinical manifestation of the cancer. From a clinical point of view, PG is of variable expression, but there are four major forms: ulcerous, pustular, bullous, and vegetative [12]. However, of all of these forms, the paraneoplastic form, which is the most frequent, is ulcerous, as was the case in our patient [11]. The pathophysiological mechanism of paraneoplastic PG remains debatable and is not yet well understood. Some suggest a hapten common to the tumor and the dermis responsible for a cross-immune reaction against the dermis [13, 14]. For Adachi *et al.* [15], this is an abnormal immune surveillance that can result in neutrophilic dysfunction, chemotaxis defects, or hyperreactivity.

The prognosis of PG is marked by a long-term evolution that remains unpredictable [1]. The relapse rate is high for commonly used drugs: 70% for prednisolone and 66% for cyclosporine [16]. Some studies have even reported a mortality rate of more than 30% [17]. In the case of paraneoplastic PG, the evolution seems to depend on the tumor, as suggested by our observation marked by a recurrence of PG lesions and their healing after the excision of the tumor, reinforcing the hypothesis of a cross-immune reaction against the dermis. This evolutionary dependence of PG on the tumor provides an opportunity for surveillance as an element of recurrence or evolution of the tumor in the event of reappearance. Furthermore, although few cases of paraneoplastic PG in colon cancer have been reported, the possibility of early appearance even before the clinical manifestation of PG may allow early diagnosis of colorectal cancer. Colonoscopy for neoplastic lesions should therefore be part of the etiological assessment in cases of PG in a patient at risk.

### Limitations

For technical reasons, immunofluorescence, although it has no diagnostic or pathogenic value, was not performed. Immunofluorescence should show immunoglobulin M and C3 deposits around the vessels at the dermal-epidermal junction.

## Conclusion

PG may be associated with rectal adenocarcinoma in paraneoplastic syndrome. In subjects at risk, early consideration is necessary, and a colonoscopy should be part of the etiological assessment in order not to miss colorectal cancer, especially when it is the recurrent ulcerative form.

## Data Availability

The authors possess the extracted data, which is available for sharing on request.

## Abbreviation

PG: Pyoderma gangrenosum
